# Optimal management of older people with frailty non-weight bearing after lower limb fracture: a scoping review

**DOI:** 10.1093/ageing/afab071

**Published:** 2021-05-17

**Authors:** Saleh Aloraibi, Vicky Booth, Katie Robinson, Eleanor Katharine Lunt, Deborah Godfrey, Alan Caswell, Margaret Kerr, Benjamin Ollivere, Adam Lee Gordon, J R F Gladman

**Affiliations:** University of Nottingham, Nottingham, UK; University of Nottingham, Nottingham, UK; NIHR Nottingham Biomedical Research Centre (BRC), Nottingham, UK; Nottingham University Hospitals NHS Trust, Nottingham, UK; University of Nottingham, Nottingham, UK; NIHR Nottingham Biomedical Research Centre (BRC), Nottingham, UK; Nottingham University Hospitals NHS Trust, Nottingham, UK; University of Nottingham, Nottingham, UK; NIHR Nottingham Biomedical Research Centre (BRC), Nottingham, UK; Nottingham University Hospitals NHS Trust, Nottingham, UK; Nottinghamshire Healthcare NHS Trust, Nottingham, UK; University of Nottingham, Nottingham, UK; Member of PPI, University of Nottingham, Nottingham, UK; University of Nottingham, Nottingham, UK; NIHR Nottingham Biomedical Research Centre (BRC), Nottingham, UK; Nottingham University Hospitals NHS Trust, Nottingham, UK; University of Nottingham, Nottingham, UK; NIHR Nottingham Biomedical Research Centre (BRC), Nottingham, UK; NIHR Applied Research Collaboration (ARC) East Midlands, Nottingham, UK; University Hospitals of Derby and Burton NHS Foundation Trust, Derby, UK; University of Nottingham, Nottingham, UK; NIHR Nottingham Biomedical Research Centre (BRC), Nottingham, UK; Nottingham University Hospitals NHS Trust, Nottingham, UK; NIHR Applied Research Collaboration (ARC) East Midlands, Nottingham, UK

**Keywords:** Optimal management, Fragility fracture, Older adults, Non-weight bearing, Scoping review

## Abstract

**Background:**

Patients with lower limb fractures who are non-weight bearing are at risk of the complications of the associated immobility and disability, particularly people with frailty, but there is lack of clarity about what constitutes optimal care for such patients. A scoping literature review was conducted to explore what evidence is available for the management of this patient group.

**Methods:**

MEDLINE (PubMed) CINAHL, EMBASE and the Cochrane databases of published literature and the HMIC and SIGLE sites for grey literature were searched for primary research studies and expert reports, using an iterative approach initially including the key term ‘non-weight bearing’. All study types were included. Analysis was by narrative synthesis.

**Results:**

No papers were identified from a search using the key phrase ‘non-weight bearing’. With this term removed, 11 indirectly relevant articles on lower limb fractures were retrieved from the searches of the electronic databases comprising three observational studies, five non-systematic review articles, a systematic review, an opinion piece and a survey of expert opinion that had relevance to restricted weight bearing patients. The observational studies indicated depression, cognition and nutrition affect outcome and hence have indirect relevance to management. The non-systematic reviews articles emphasised the importance of maintaining strength and range of movement during immobilisation and advised an orthogeriatric model of care. Fourteen UK and 97 non-UK guidelines relevant to fragility fractures, falls and osteoporosis management were found in the grey literature, but none made specific recommendations regarding the management of any period of non-weight bearing.

**Discussion:**

These findings provide a summary of the evidence base that can be used in the development of a clinical guideline for these patients but is not sufficient. We propose that, a guideline should be developed for these patients using an expert consensus process.

## Key points

- We found no research literature specific to the care of older people with or without frailty, and little related to weight-bearing restrictions, after a lower limb fracture.
- Observational studies show that depression, cognition and nutrition affect outcomes in patients with weight-bearing restrictions.
- Non-systematic review articles emphasise the importance of maintaining strength and range of movement during immobilisation.
- There are many guidelines indirectly relevant to this patient group that concern the management of their osteoporosis and falls prevention but do not mention non-weight bearing.

## Introduction

Lower limb fractures rise in incidence with age and one-third of all orthopaedic trauma patients are treated for lower extremity fractures [1]. In a UK-based epidemiological study the annual incidence of lower limb fractures across all ages was 2.9/1000 for men and 3.4/1000 for women, rising to 3.2/1000, 11.9/1000 and 22/1000 in those the aged 70–79, 80–89 and above 90, respectively [2].

Depending upon the fracture site, its surgical treatment and the underlying bone health, the orthopaedic surgeon advises on the preferred weight bearing status to enable bone healing: non-weight bearing (NWB); partial-weight bearing (PWB); or full weight bearing (FWB) [3, 4]. Lower limb fractures, whether surgically treated or not, can require a period of weight-bearing restrictions before returning to FWB for 6–12 weeks [5, 6]. In patients with frailty, comorbidity or limited prior mobility, being NWB can lead to immobility, dependency and lengthy hospital admissions [7, 8]. Associated complications of this include bone loss [9], muscle loss [10], muscle function and muscle strength loss [11], potentially leading to a spiral of increasing frailty, falls and further fractures [12]. The judgment to advise NWB balances these competing risks.

Optimal care of patients with frailty who are NWB after a lower limb fracture would minimise the risks due to immobility and optimise bone healing. However, there is lack of clarity about what constitutes such optimal care. To help provide this clarity, we conducted a scoping literature review to explore the evidence available for the management of this patient group. Our research question was, ‘What evidence is available for the optimal care and management of older people who are non-weight bearing after lower limb fracture?’

## Method

### Protocol and registration

Our scoping review protocol in this study pre-defined the objectives, methods and reporting of the review and allowed for transparency of process. This protocol was published [13]. The Preferred Reporting of Items for Systematic Reviews and Meta-Analyses—extension for scoping reviews (PRISMA-ScR) Statement [14] was followed.

### Eligibility criteria

We sought published material about the care of older people with or without frailty with lower limb fractures (pelvic/hip region, femur, patella, tibia/fibula, ankle, foot, toes) who require a period of NWB.

We deemed all evidence types (meta-analyses, systematic reviews, randomised controlled studies, cross-sectional studies, case control studies, case-series studies, cohort studies, qualitative studies, other reviews and expert commentaries) and clinical guideline reports to be of potential relevance, and accordingly searched both electronic databases of published material and the grey literature.

To be specific to our study group of interest, we excluded material related to: lower limb fractures due to high velocity trauma, polytrauma or studies about athletes; and study groups aged less than 65. For convenience, we excluded material not published in English.

### Information sources

To ensure a comprehensive search of the published research literature, the following databases were searched for articles: MEDLINE (PubMed) CINAHL, EMBASE and the Cochrane database.

Grey literature searching for guidelines took place using online sources designed specifically for clinical guidelines such as NICE Guidelines in the UK, the International Guideline Network, International Guideline Library, NICE Guidelines Australia and World Health Organization Clinical Practice Guidelines websites. Google Scholar was also utilised to identify any other primary sources within grey literature. The searches were undertaken between September and December 2019.

### Search strategy

We used identical approaches and terms for searches of the electronic databases and grey literature.

The following search terms were used initially: geriatric, older person, older people, aged, optimal care, best care, care pathway, trauma, NWB and lower limb fractures assessment, outcome, results, rehabilitation and immobilisation. Each of the search terms were combined with these keywords: ‘non-weight bearing’ and ‘lower limb fracture’ and with each of the following three keywords: optimal care, management best care and intervention.

The first search including the term ‘non-weight bearing’ did not identify any studies that met the inclusion criteria for this review. To broaden the scope, in the second search removing that term on the basis that the NWB patient group is a subset of those with lower limb fragility fractures, we found research papers and review articles about lower limb fragility fractures referring to ‘weight bearing restrictions’ (PWB and NWB) from the electronic databases of published articles, and a large number of national and international reports and guidelines from or on behalf of professional societies from the grey literature search about lower limb fragility fractures and their management. This literature was classified according to country or global region.

### Selection of sources of evidence

One reviewer (Saleh Aloraibi) independently screened the titles and abstracts of citations according to the eligibility criteria. Then, the selection was sent to initially one reviewer (John Gladman) who refined and confirmed the selection. Then these selections were sent to another reviewer (Vicky Booth) to confirm the selection that met the criteria for inclusion.

### Data charting process

Data extraction table was created to collect data from each study including date and country in which study was conducted, the type of participants, their age, the sample size and settings. The results and implications of each paper relevant to our research question were extracted where relevant to any of the following professional groups (medicine, orthopaedic, nursing, physiotherapy, occupational therapy, dietetics, pharmacy), or of more generic relevance (patients, family, team working). Data were extracted by one reviewer (Saleh Aloraibi) and then checked by two others (John Gladman & Vicky Booth) and discussed until there was agreement.

### Critical appraisal of individual sources of evidence

In view of the nature of this scoping review and the evidence found, no formal critical appraisal of the literature was conducted**.**

### Synthesis of results

The summary of the findings was tabulated. A narrative synthesis summarising the findings was undertaken, seeking to describe the evidence available and identify the gaps in the current literature base.

For clinical guidelines, as we found a large number of non–UK-based clinical guidelines related to fragility fractures, we chose briefly to summarise the content of the UK guidelines and present the references for those from the rest of the world as Appendix 1 in the supplementary data.

## Results

### Selection of evidence

**Figure 1:**
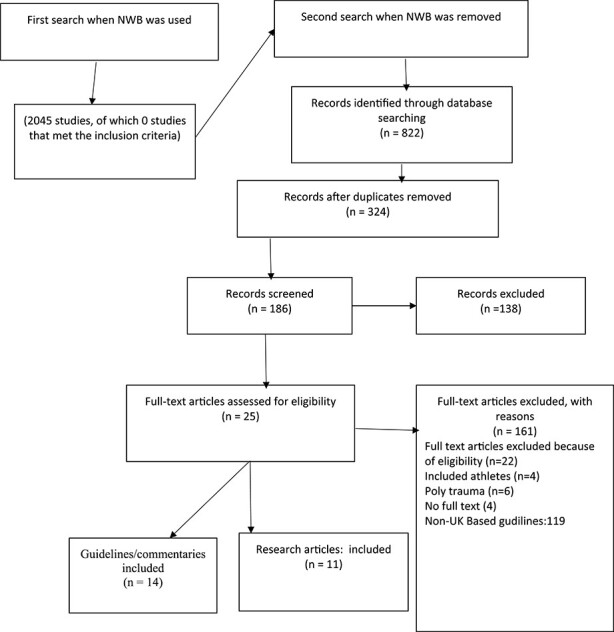
PRISMA flow diagram for scoping review process.

Figure 1 summarises the search and selection process for the evidence used in this review. It confirms the absence of specific literature using the phrase ‘non-weight bearing’. It also shows that when we broadened the scope of our review we found many articles and guidelines about lower limb and fragility fractures. Eleven articles were found from the electronic databases, and 14 UK guidelines and 97 non-UK guidelines from the grey literature. Only three guidelines were found in the electronic database searches.

### Characteristics of the evidence

Table 1 summarises the characteristics of the 11 articles found from the search of electronic databases [7, 15–24]. Three observational cohort studies, five non-systematic review articles, one systematic review, one survey of expert opinion and one opinion piece was found. The three observational studies (ranging from 8 to 1746 participants) were from Australia, Malaysia and USA, and concerned lower limb fractures with ‘restricted weight bearing’ which included patients with partial and NWB management. Three of the five non-systematic review articles (expert opinion articles) described the management of patients with lower limb fractures and two concerned ankle fractures. One systematic review and one review paper concerned venous thromboembolism (VTE) prophylaxis in patients with a lower limb cast and lower limb immobility. The survey of expert opinion asked orthopaedic surgeons about their choice of duration of periods of NWB and factors affecting their choice. The opinion piece was a description of the development of a fracture liaison service. In none of these papers was there a description of the specific management of patients who are NWB—other than that venous thrombo-prophylaxis is likely to be important, and regarding the duration of the period of NWB.

**Table 1:** Articles retrieved from the electronic database searches

First author, date, reference number	Country	Fracture sites	Participants	Age	Gender	Sample size	Study design	Setting
Brown 2019 [15]	Australia	Lower limb and multi-trauma	Orthopaedic patients admitted with weight-bearing restrictions after lower limb fracture	Mean 71	31% male	111	Retrospective 24 month observational study	Hospital (level 4 subacute rehabilitation facility)
Gitajn 2018 [16]	USA	Lower limb	Older adults admitted with lower extremity orthopaedic injuries	≥65	48% male	1746	Retrospective 120 month observational study	Hospital (level 1 trauma centre)
Ibrahim 2019 [17]	Malaysia	Lower body	Adults	≥50 (69.62 mean ± 10.68, *n* = 57 ≥ 65,	38% male	89	Prospective 24 month observational study	Hospital (orthopaedic wards at ×2 hospitals/sites
Strauss 2007 [18]	USA	Ankle	Geriatric patients	n/a	n/a	n/a	Non-systematic review	Not specified
Hoffmeyer 2019 [19]	Switzerland	Pelvic ring and lower limbs	Older patients	n/a	n/a	n/a	Non-systematic review	Not specified
Rammelt 2016 [20]	Germany	Ankle	Older patients	n/a	n/a	n/a	Non-systematic review	Not specified
Horner 2020 [21]	UK	Lower limb	Adult patients with lower limb immobilisation	n/a	n/a	n/a	Non-systematic review	Hospital
Kadakia 2017 [22]	USA and Israel	Ankle	Geriatric patients	n/a	n/a	n/a	Non-systematic review	Not specified
Hickey 2018 [23]	UK	Foot and ankle	Adult patients with below knee cast treatment	n/a	n/a	n/a	Systematic review and meta-analysis	Hospital and community
Mitchell 2011 [24]	UK	Lower limb	Older patients with fragility fractures	≥50	n/a	n/a	Invited opinion paper	Hospital—Fracture Liaison Service (FLS).
Swart 2015 [7]	USA	Ankle	Non-weight bearing patients	n/a	n/a	n/a	Cross-sectional expert opinion survey	Hospital

Table 2 summarises the characteristics of the 14 relevant UK guidelines. Seven of the UK guidelines or expert reports primarily concerned the management of osteoporosis [25, 32] and seven [33–39] were more generic and concerned fragility fracture management including not only the management of osteoporosis, but also the preventions of falls, and integrated services to deliver both of these (Fracture Liaison Services).

**Table 2:** UK-based clinical guidelines for fragility fractures and osteoporosis

Year of publication, reference number	Title	Setting	Publishing organisation	Patient group	Intended audience	Content
Primarily osteoporosis						
2008 [25]	Raloxifene for the primary prevention of osteoporotic fragility fractures in post-menopausal women	Hospital and community	NICE	Post-menopausal women	Health care professionals Patients Commissioners and/or providers	80 pages (guidance recommendations and how to implement it)
2018 [27]	Raloxifene and teriparatide for the secondary prevention of osteoporotic fragility fractures in post-menopausal women	Hospitals and community	NICE	Post-menopausal women	Health care professionals Patients Commissioners and/or providers	85 pages of guidance recommendations and how to implement them
2015 [28]	SIGN 142: Management of osteoporosis and the prevention of fragility fractures	Hospital and community	SIGN-Scotland	Adults with osteoporosis	Rheumatologists Endocrinologists General practitioners Geriatricians Orthopaedic surgeons Gynaecologists specialist Osteoporosis nurses Pharmacists	128 pages of recommendations related to the management of osteoporosis and the prevention of fragility fractures.
2017 [29]	Osteoporosis Quality Standards	Hospitals and community	NICE	Adults with or at risk of fragility fracture Adults with a history of falls Adults treated for osteoporosis	Service providers Healthcare professionals Service commissioners	4 standards
2018 [30]	Impact falls and fragility fractures	Hospital and community	NICE	People with osteoporosis	Health professionals	20 pages of commentaries and recommendations
2012 [31]	Osteoporosis: assessing the risk of fragility fracture	Hospital and community	NICE	People with osteoporosis at risk of fragility fracture	Local commissioners and providers of healthcare	17 standards and substandards—14 pages
2019 [32]	Osteoporosis: assessing the risk of fragility fracture	Hospital and community	National Clinical Guideline Centre	People with osteoporosis at risk fragility fracture	All healthcare professionals and other staff who care for people at risk of fragility fracture	18 standards and recommendations (97 pages)
General						
2007 [33]	The care of patients with fragility fracture (Blue Book)	Hospitals	British Orthopaedic Association	Older people with fragility fractures	Health professionals Health service managers	6 standards with 80 pages of recommendations
2012 [34]	Guidelines for the Physiotherapy management of older people at risk of falling	Hospital and community	AGILE: Chartered physiotherapists working with older people	Old people who are at risk of falling	Physiotherapists for older people and the multidisciplinary rehabilitation team	4 standards, in 8 pages
2012 [35]	Right Care Pathway: Falls and Fragility Fractures	Hospital and community	NHS Right care, Public Health England, National Osteoporosis Society	Adults with history or at risk of falls	Health professionals Commissioners Patients	34 pages of recommendations and commentaries
2013 [36]	Assessment and prevention of falls in older people	Hospital and community	NICE	All people aged 65 or older People aged 50 to 64 who are admitted to hospital and are judged by a clinician to be at higher risk of falling	Healthcare and other professionals and staff who care for older people who are at risk of falling	315 pages of commentaries and recommendations
2017 [37]	Quality Standards for Osteoporosis and Prevention of Fragility Fractures	Hospital and community	The Royal Osteoporosis Society-UK	Adults at increased risk of fragility fractures	Patients, carers and families Health professionals delivering osteoporosis services Health professionals involved in fragility fracture prevention Commissioners Managers	7 standards
2017 [38]	Falls and fracture consensus statement. Supporting commissioning for prevention	Hospital and community	Public Health England	Adults >65 with a history or at risk of fractures	Local commissioning and strategic leads in England with a remit for falls, bone health and healthy ageing	22 pages of commentaries and recommendations
2019 [39]	Effective Secondary Prevention of Fragility Fractures: Clinical Standards for Fracture Liaison Services	Hospital and community	Royal Osteoporosis Society	Adults >50 or older who have had a fragility fracture	Patients, their carers and families Health professionals in Fracture Liaison Services (FLS) and fracture prevention Commissioners Managers	6 standards (with 23 substandards) over 38 pages

Of the 97 guidelines from non-UK countries were, 34 were from the rest of Europe, 34 were from Asia-Pacific area, 15 were from North America, 10 were from the Middle East and Africa and four were from Latin America. Appendix 1 in the supplementary data available to subscribers in Age and Ageing online lists these [41–137]. None of the UK or non-UK guidelines specifically described management of patients during any period of NWB during their care, although they addressed the management of related issues such as reduced mobility, propensity to falls and the frailty state.

### Narrative synthesis of relevant findings from the evidence

The three observational studies [15–17] indicated that for patients with lower limb fractures who had ‘weight bearing restrictions’ (i.e. either NWB or PWB):

- Had a longer length of stay, even though 23% were discharged to their own homes or homes with friends or relatives whereas still with weight-bearing restrictions with 6% were discharged to residential care facilities [15].
- Length of stay was prolonged by depression, cognitive impairment and undernutrition [17].
- Discharge mobility was predicted by their prior mobility status before the injury [17].
- Medical complications were common [15] and people with weight-bearing restrictions had significantly higher 5 year mortality that those without weight-bearing restrictions [16].

These papers therefore provide natural history information of relevance to the management of patients who are NWB, but fall short of describing the specific management actions that might follow from these observations.

Key relevant assertions from the five non-systematic review articles were:

- It should not be assumed that all patients with ankle fractures (many of whom will be NWB) have osteoporosis: although many will and so a diagnosis should be sought—risk assessment and bone densitometry being advised [18, 20]. Many ankle fractures are not fragility fractures.
- Range of joint movement should be maintained through active and passive exercise during NWB.
- Strength should be maintained, using isometric exercise in NWB limbs.
- Diabetes, and poorly controlled diabetes in particular, is a strong risk factor for complications and should be managed closely.
- Skin complications are amongst the most serious complications (especially in open fractures) and so skin and its perfusion should be carefully assessed.
- An orthogeriatric model of care, like that used for hip fracture, for these patients was advised.

As summarised above, non-systematic reviews provided the only descriptions in the published literature of aspects of the management of these patients—drawn from clinical experience.

The systematic review of VTE prophylaxis for patients with a cast (often associated with being NWB) concluded that low molecular weight heparin reduces the incidence of symptomatic VTE [23].

The expert opinion piece about setting up a fracture liaison service largely concerned the lengthy consultation process to obtain the necessary co-operation between the multiple stakeholders involved (hospital/community, falls/osteoporosis/orthopaedic surgery). NWB was not mentioned.

The specific guidelines for osteoporosis were mainly concerned with the appropriate use specific medications for osteoporosis—anti-resorptive drugs. None made specific reference to the NWB period.

Whilst not directly addressing the period of NWB, the guidelines describing fall and fragility fracture care held the common views that:

- Whilst accepting that the immediate management of different fractures (e.g. wrist, spine, hip) is site-specific, they should be considered together as fragility fractures.
- Fragility fractures occur as a result both of falls and of poor bone health and hence that fracture services should assess both falls risk and bone health.
- (Fracture Liaison Services) should be in place to co-ordinate the identification and management of osteoporosis and the prevention of falls for patients with fragility fractures. Guidelines for such services typically specify: the identification
- Of people with fragility fracture of different types and in different settings; the co-ordination of the investigation of these patients and their fracture risk; information for patients of the lifestyle and medical interventions to reduce their risk; the co-ordination of the delivery of specific interventions (anti-resorptive medications, strength and balance exercise); the integration of these activities efficiently and their quality assurance.
- Falls prevention is multifactorial and includes the assessment and modification of risk factors the most important interventions being strength and balance training

## Discussion

We found little research literature specific to the care of older people with or without frailty who are NWB after a lower limb fracture. Observational studies of patients with weight-bearing restrictions (i.e. NWB and PWB) indicated the importance of depression, cognition and nutrition upon outcome, and thereby implied that a focus on these risk factors would be wise. Only the non-systematic reviews (expert commentaries of clinical experience and opinion) discussed the management of these patients by emphasising the importance of identifying osteoporosis, skin care, diabetes management, maintaining strength and range of movement during immobilisation, and advising an orthogeriatric model of care. We found abundant literature in terms of clinical guidelines about the management of fragility fractures in general: these mainly covered the identification and management of osteoporosis, and the assessment and management of falls risk, but did not address NWB specifically.

The scoping review approach taken was sufficient to identify the extremely limited extent of specific literature on this patient group. We appreciate that it could be extended were we to have pursued further guidance for the care of older people with frailty in general, such as the management of pain, incontinence, specific nutritional guidance of hospitalised patients, the use of weight bearing versus NWB exercise [40], and so on, but we considered this too far out of scope. We acknowledge that grey literature searching is notoriously difficult and that we could have missed valuable reports of expert opinion similar to the non-systematic reviews that we did find—for example, those published in languages other than English or those held on private websites. Nevertheless, given that we found so little specific information related to the care of patients who are NWB after lower limb fracture, we believe that this work is an important contribution to the process of developing guidelines for their optimal care.

Such guidelines should not only take account of the limited findings summarised here, but should also accommodate existing generic basic care requirements for older people with frailty such as pain and continence care, and include common sense matters such as ensuring clarity about the nature, duration, monitoring and implications of a period of NWB. Further work to obtain expert opinion from those who routinely provide care for these patients would be of value. Such an exercise could identify whether there is consensus about specific management practices such as nutritional interventions, exercises that can be done when NWB, or the situations when anti-resorptive treatment can be given without prior bone mineral density assessment. Using the information from our review and using a consensus guideline development process it should be possible to develop a useful clinical guideline for this patient group, even though it would not be based upon robust trial evidence.

## Supplementary Material

aa-20-1469-File002_afab071
