# Supplementary material for: Optimal management of older people with frailty non-weight bearing after lower limb fracture: a scoping review
Source: Age Ageing. 2021 May 17;50(4):1129–36. doi: 10.1093/ageing/afab071 (PMC8266651; doi:10.1093/ageing/afab071)
Supplement: aa-20-1469-File002_afab071 [file aa-20-1469-file002_afab071.docx]

Optimal management of older people with frailty non-weight bearing after lower limb fracture: A scoping review

**SUPPLEMENTARY DATA**

**Appendix 1.** Non-UK based guidelines and policies for fracture liaison systems & osteoporosis guidelines

**THE REST OF EUROPE**

1. Kanis, J., Cooper, C., Rizzoli, R. et al. European guidance for the diagnosis & management of osteoporosis in postmenopausal women. Osteoporos Int (2018). <https://doi.org/10.1007/s00198-018-4704-5>.
2. Kanis JA. European Society for Clinical and Economic Aspects of Osteoporosis and Osteoarthritis (ESCEO). European guidance for the diagnosis and management of osteoporosis in postmenopausal women. Osteoporosis Int. 2008;19:399-428.
3. Kanis, J.A., Cooper, C., Rizzoli, R. et al. Executive summary of European guidance for the diagnosis and management of osteoporosis in postmenopausal women. Calcif Tissue Int (2019). <https://doi.org/10.1007/s00223-018-00512->
4. Blain H, Masud T, Dargent-Molina P, Martin FC, Rosendahl E, van der Velde N, Bousquet J, Benetos A, Cooper C, Kanis JA, Reginster JY. A comprehensive fracture prevention strategy in older adults: the European Union Geriatric Medicine Society (EUGMS) statement. The journal of nutrition, health & aging. 2016 Jun 1;20(6):647-52.
5. World Health Organization, World Health Organization. Ageing, Life Course Unit. WHO global report on falls prevention in older age. World Health Organization; 2008.
6. Dimai HP, Pietschmann P, Resch H, Leb G, Klaushofer K. Leitfaden zur medikamentösen Therapie der postmenopausalen Osteoporose. Wien Med Wschr 2002; 152: 596-612.
7. Gielen E, Bergmann P, Bruyère O, Cavalier E, Delanaye P, Goemaere S, Kaufman JM, Locquet M, Reginster JY, Rozenberg S, Vandenbroucke AM. Osteoporosis in frail patients: a consensus paper of the Belgian bone club. Calcified tissue international. 2017 Aug 1;101(2):111-31.
8. Body JJ, Bergmann P, Boonen S, Boutsen Y, Devogelaer JP, Goemaere S, Kaufman JM, Rozenberg S, Reginster JY. Evidence-based guidelines for the pharmacological treatment of postmenopausal osteoporosis a consensus Osteoporosis International 21: 1657–1680, 2010.
9. Devogelaer JP, Goemaere S, Boonen S, Body JJ, Kaufman JM, Reginster JY, Rozenberg S, Boutsen Y. Evidence-based guidelines for the prevention and treatment of glucocorticoid-induced osteoporosis, Osteoporosis International 17: 8-19, 2006.
10. Borissova A-M, Zacharieva S, Boyanov M, Kovacheva R, Rashkov R, Kolarov Z, Popivanov P, Shinkov A, Petranova T, Ed. Prof. Anna-Maria Borissova. Recommendations for good clinical practice in osteoporosis of Bulgarian Society of Endocrinology and Bulgarian Society of Rheumatology. SD Simolini.94, Sofia, 2013.
11. Borissova A-M, Boyanov M, Kolarov Z, Popivanov P, Shinkov A, Stoilov R, Svinarov D. Petranova T, Pilossof V. Ed. Prof. Anna-Maria Borissova Recommendations for the diagnosis, prevention and treatment of vitamin D insufficiency and deficiency of Bulgarian Society of Endocrinology, Bulgarian Society of Rheumatology, Bulgarian Pediatric Association, Bulgarian Society of Clinical Laboratory. SD Simolini 94, Sofia, 2013.
12. Rosa J, Šenk F, Palička V; with cooperation of Blahoš J, Bayer M., Broulík P, Horák P, Kasalický P, Kučerová I, Kuba V, Kutílek Š, Pikner R, Vyskočil V. Diagnosis and treatment of postmenopausal osteoporosis. Statement of the Czech Society for Metabolic Bone Diseases/Osteol Bull 2015; 20/4/:150-168 .
13. Rosa J, with cooperation of Blahoš J, Bayer M., Broulík P, Horák P, Kasalický P, Kučerová I, Kuba V, Kutílek Š, Palička V, Pikner R, Šenk F, Vyskočil V.Osteoporosis in males. Statement of the Czech Society for Metabolic Bone DiseasesOsteol Bull 2016; 21/2/:42-48.
14. Briot K, Cortet B, Thomas T, Audran M, Blain H, Breuil V, Chapuis L, Chapurlat R, Fardellone P, Feron JM, Gauvain JB, Guggenbuhl P, Kolta S, Lespessailles E, Letombe B, Marcelli C, Orcel P, Seret P, Trémollières F, Roux C. Update of French guidelines for the pharmacological treatment of postmenopausal osteoporosis. Joint Bone Spine. 2012 May;79(3):304-313. PubMed ID 22521109.
15. Makras P1, Vaiopoulos G, Lyritis GP. 2011 Guidelines for the Diagnosis and Treatment of Osteoporosis in Greece. J Musculoskelet Neuronal Interact. 2012 Mar;12(1):38-42.; Greek National Medicine Agency.http://www.ismni.org/jmni/pdf/47/06MAKRAS.
16. Cianferotti L, Brandi ML. Guidance for the diagnosis, prevention and therapy of osteoporosis in Italy. Clinical cases in mineral and bone metabolism. 2012 Sep;9 (3):170.
17. M. Rossini, S. Adami, F. Bertoldo, D. Diacinti, et al. Guidelines for the diagnosis, prevention and management of osteoporosis 2016.Reumatismo: Official Journal of the Italian Soc. for Rheumatology. (SIOMMS), Vol 68, No 1 (2016)http://dx.doi.org/10.4081/reumatismo.2016.870 .
18. Nuti R, Brandi ML, Checchia G, Di Munno O, Dominguez L, Falaschi P, Fiore CE, Iolascon G, Maggi S, Michieli R, Migliaccio S. Guidelines for the management of osteoporosis and fragility fractures. Internal and emergency medicine. 2019 Jan 24;14(1):85-102.
19. Lejnieks Aivars, Aizstrauta Tamāra, Ādamsone Ināra, Matule Dace, Rasa Ingvars,Rumba Ingrīda, Platkājis Ardis, Vestermanis Viktors. Osteoporozes diagnostikas, profilakses un ārstēšanas vadlīnijas. Rīga, 2005.
20. Rasa Ingvars , Ādamsone Ināra, Daukste Ilze, Pavliņa Inese, Platkājis Ardis, Vētra Anita, Zelča Signe. Osteoporosis clinical guidelines. Rīga: Latvijas Osteoporozes un kaulu metabolo slimību asociācija, 2013/2014 (project)Lithuania Guideline for osteoporosis diagnosis and treatment.
21. Alekna V, Tamulaitienė M, Krasauskienė A. Osteoporozės diagnostikos ir gydymo metodika. Nauja 2011 metų redakcija (Guideline for osteoporosis diagnosis and treatment.Gerontologija. 2011;12(2):110-130.http://www.gerontologija.lt/files/edit_files//File/pdf/2011/nr_2/2011_110_130.pdf.
22. Richtlijn Osteoporose en fractuurpreventie derde herziening (2011), Nederlandse Vereniging voor Reumatologiehttps://www.nvr.nl/wp content/uploads/2014/11/CBO-richtlijn-osteoporose-en-fractuurpreventie-2011.
23. Tuinhout M, van Roermund PM. Richtlijn Osteoporose en fractuurpreventie. PodoSophia. 2015 Mar 1;23(2):23-5.
24. Faglige retningslinjer for forebygging og behandling av osteoporose og osteoporotiske brudd. 12/2005IS-1322 ISBN978-82-8081-076-5 Utgitt av: Sosial- og helsedirektoratet Kontakt: Avdeling for primærhelsetjenesterwww.shdir.no.
25. Lorenc R, Gluszko P, Franek E, Jablonski M, Jaworski M, Kalinka-Warzocha E, et al. Guidelines for the diagnosis and management of osteoporosis in Poland. Update 2017 . Endokrynologia Polska, DOI: 10.5603/EP.2017.0062, Tom/Volume 68; Numer/Number 5/2017 ISSN 0423–104X.
26. Portuguese/Society of Rheumatology and the Portuguese Society of Metabolic Bone Diseases (SPODOM). Recommendations for the Diagnosis and treatment of Osteoporosis.2007/http://www.spreumatologia.pt/files/guideline/18_recomenda_es_para_o_diagn_stico_e_terap_utica_da_osteoporose.
27. Slovak Republic Ministry of Health. Guidelines for the Diagnosis and Treatment of Patients with Osteoporotic Fractures/Ministry of Health Bulletin, February 4, 2011, No. 59, Part 1-3/.
28. Slovak Republic Ministry of Health. Guidelines for the Diagnosis of Glucocorticoid-induced Osteoporosis Ministry of Health Bulletin, November 13, 2009, No. 57, Part 51-53 English Translation.
29. Slovak Republic Ministry of Health. Guidelines for the Diagnosis and Treatment of Osteoporosis Ministry of Health Bulletin, March 1, 2006, No.
30. Smernice za odkrivanje in zdravljenje osteoporoze. Guidelines for the detection and treatment of osteoporosis (2013).
31. Kocjan T, Preželj J, Pfeifer M, Jensterle Sever M, Čokolič M, Zavratnik A, Vestn Z. 2013; 82: 207http://vestnik.szd.si/index.php/ZdravVest/article/view/635.
32. Spanish Society for Research on Bone and Mineral Metabolism .Clinical practice guidelines for postmenopausal, glucocorticoid-induced and male osteoporosis. Spanish Society for Research on Bone and Mineral Metabolism (3rd updated version 2014) <http://www.revclinesp.es/es/linkresolver/guias-practica-clinica-osteoporosis-posmenopausica/S0014256515002192/.>
33. González Macías J, Guañabens Gay N, Gómez Alonso C, del Río Barquero L, Muñoz Torres M, Delgado M, et al. Carranza Guías de práctica clínica en la osteoporosis posmenopáusica, glucocorticoidea y del varón. Sociedad Española de Investigación Ósea y del Metabolismo Mineral (SEIOMM). 2008, (Comité de Redacción, en representación del Comité de Expertos de la SEIOMM para la elaboración de las Guías). Download from [www.seiomm.org](http://www.seiomm.org).
34. Socialstyrelsens riktlinjer för vård och behandling av höftfraktur. National guidelines for management of hip fractures) HBWH 2003/ ISBN: 91-7201-758-9; Treatment recommendations from the MPA 2007/.[www.lakemedelsverket.se/Tpl/RecommendationsPage.aspx?id=2605](http://www.lakemedelsverket.se/Tpl/RecommendationsPage.aspx?id=2605).

**ASIA-PACIFIC**

1. Dent E, Lien C, Lim WS, Wong WC, Wong CH, Ng TP, Woo J, Dong B, de la Vega S, Poi PJ, Kamaruzzaman SB. The Asia-Pacific clinical practice guidelines for the management of frailty. Journal of the American Medical Directors Association. 2017 Jul 1;18(7):564-75.
2. Department of Health, State of Western Australia. Osteoporosis Model of Care Musculoskeletal, Diabetes & Endocrine, Falls Prevention and Aged Care Health Networks (Osteoporosis Model of Care). 2011.
3. National Health and Medical Research Council. Clinical guideline for the prevention and treatment of osteoporosis in postmenopausal women and older men. February 2010. ISBN 978-0-86906-306-4. http://www.racgp.org.au/Content/NavigationMenu/ClinicalResources/RACGPGuidelines/osteoporosis.
4. Department of Health, State of Western Australia. Orthogeriatric Model of Care. 2008.
5. Queensland Government. Falls prevention best practice guidelines for public hospitals and state government residential aged care facilities incorporating a community integration supplement. Queensland, Australia; 2003. Available from: www.health.qld.gov.au/fallsprevention/best_practice/falls.
6. Australian Council for Safety and Quality in Health Care. Preventing falls and harm from falls in older people: best practice guidelines for Australian hospitals and residential aged care facilities. Available from NSW Falls Injury Prevention Network:[www.powmri.edu.au/fallsnetwork/safety_and_quality_council_docum.htm 60](http://www.powmri.edu.au/fallsnetwork/safety_and_quality_council_docum.htm%2060).
7. Chris Baggoley SL, Jaqueline C, Mandy H, Keith H, Kirsten H, Lorraine L, Rozelle W. Preventing Falls and Harm From Falls in Older People.2009.
8. Osteoporosis and Bone Mineral Society of the Chinese Medical Association .Guidelines for primary osteoporosis diagnosis and management, Osteoporosis and Bone Mineral Society of the Chinese Medical Association 2017. <http://kns.cnki.net/kcms/detail/13.1222.R.20171027.1618.002.html>.
9. Orthopedic Society of Chinese Medical Association .Guidelines for osteoporosis fracture management,. Chin J Otthop, 2008; 28(10):875-878.
10. Chinese Medical Association. Osteoporosis Treatment Guidelines, 2006, with an update in 2011  <http://www.haodf.com/zhuanjiaguandian/liaodefa_583581080.htm>.
11. [The Application Guideline for Vitamin D and Bone Health in Chinese Adult](https://www.iofbonehealth.org/sites/default/files/media/PDFs/National%20Guidelines/2014-China_Vitamin_D_guidelines.pdf) (2014 Standard Edition). Chin J Osteoporos, September 2014, Vol 20, No. 9. Published online [www.wanfangdate.com.cn](http://www.wanfangdate.com.cn/) doi:10.3969/j.
12. Cheung SK, Cheung TC, Choi TC, Chow SL, Ho YY, et al. Guideline for Clinical Management of Postmenopausal Osteoporosis in Hong Kong’, Hong Kong Med J, vol. 19, . <http://www.hkmj.org/supplements/article_pdfs/hkm1304sp2p6.pdf>.
13. Krishnamurthy V, Sharma A, Aggarwal A, Kumar U, Amin S, Rao UR, et al. ‘Indian Rheumatology Association Guidelines for Management of Glucocorticoid-induced Osteoporosis’, Indian J Rheumatol, 2011, vol. 6, no. 2, pp. 68-75..
14. LMeeta; Harinarayan, Raman Marwah, Rakesh Sahay, Sanjay Kalra, Sushrut Babhulkar. Clinical practice Guidelines on postmenopausal osteoporosis: An executive summary and recommendations .Indian Menopause Society
    [Update 2019](http://worldosteoporosisday.org/Indian-Menopause-Society-Update-Guidelines-Postmeno-OP-2019.pdf).
15. Nawata H, Soen S, Takayanagi R, Tanaka I, Takaoka K, Fukunaga M, Matsumoto T, Suzuki Y, Tanaka H, Fujiwara S, Miki T. Guidelines on the management and treatment of glucocorticoid-induced osteoporosis of the Japanese Society for Bone and Mineral Research. Journal of bone and mineral metabolism. 2005 Mar 1;23(2):105-9.
16. Orimo H, Nakamura T, Hosoi T, Iki M, Uenishi K, Endo N, et al. guidelines for prevention and treatment of osteoporosis.
    Arch Osteoporos (2012) 7:3-20
    <http://link.springer.com/article/10.1007/s11657-012-0109-9>
17. Suzuki Y, Nawata H, Soen S. et al. Guidelines on the management and treatment of glucocorticoid-induced osteoporosis of the Japanese Society for Bone and Mineral Research: 2014 update
    J Bone Miner Metab (2014) 32: 337. doi:10.1007/s00774-014-0586-6
    <http://link.springer.com/article/10.1007%2Fs00774-004-0596-x>
18. Nakamura T. Absolute risk for fracture and WHO guideline. Fracture risk assessments recommended by World Health Organization and Japanese guidelines for prevention and treatment of osteoporosis 2006. Clinical calcium. 2007 Jul;17(7):1022-8.
19. Suzuki Y. Glucocorticoid and Bone. Updated Japanese guidelines for the management of glucocorticoid-induced osteoporosis. Clinical calcium. 2014 Sep;24(9):1309-18.
20. Nojiri S, Burge RT, Flynn JA, Foster SA, Sowa H. Osteoporosis and treatments in Japan: management for preventing subsequent fractures. Journal of bone and mineral metabolism. 2013 Jul 1;31(4):367-80.
21. Nishizawa Y, Ohta H, Miura M, Inaba M, Ichimura S, Shiraki M, et al. Guidelines for the use of bone metabolic markers in the diagnosis and treatment of osteoporosis . Journal of bone and mineral metabolism. 2013 Jan 1;31(1):1-5.
22. Kim KI, Jung HK, Kim CO, Kim SK, Cho HH, Kim DY, et al. Evidence-based guidelines for fall prevention in Korea. The Korean journal of internal medicine. 2017 Jan;32(1):199.
23. Jung D, Shin S, Kim H. A fall prevention guideline for older adults living in long‐term care facilities. International nursing review. 2014 Dec;61(4):525-33.
24. Park SY, Gong HS, Kim KM, Kim D, Kim H, Jeon CH, et al. Korean guideline for the prevention and treatment of glucocorticoid-induced osteoporosis. Journal of Rheumatic Diseases. 2018 Oct 1;25(4):263-95.
25. Malaysian Osteoporosis Society. Clinical Guidance on Management of Osteoporosis.2012. available on the following websites [www.osteoporosis.my](http://www.osteoporosis.my/) ; [www.moh.gov.my](http://www.moh.gov.my/); [www.acadmed.org.my](http://www.acadmed.org.my/); [www.msr.my](http://www.msr.my/).
26. Amir SK, Chan SP, Abdullah BJ. Clinical Practice Guidelines on Management of Osteoporosis. Clinical practice guidelines on management of osteoporosis (2006).
27. Yeap SS, Hew FL, Lee JK, Goh EM, Chee W, Mumtaz M, et al. The Malaysian Clinical Guidance on the management of postmenopausal osteoporosis. International journal of rheumatic diseases. 2013 Feb;16(1):30-40.
28. Yeap SS, Hew FL, Damodaran P, Chee W, Lee JK, Goh EM, et al. A summary of the Malaysian Clinical Guidance on the management of postmenopausal and male osteoporosis. Osteoporosis and sarcopenia. 2016 Mar 1;2(1):1-2.
29. Perez EC, Canete A, Bonificio L, Llamado LQ, Martinez R, Li-Yu J , et al. Consensus statements on osteoporosis diagnosis, prevention, and management in the Philippines International Journal of Rheumatic Diseases. Vol 14, Issue 3, Aug. 2011, p. 223–238.<http://onlinelibrary.wiley.com/doi/10.1111/j.1756-185X.2011.01626.x/abstract>.
30. Singapore Ministry of Health. Clinical Practice Guidelines for Osteoporosis 3/2008, January 2009. Available from:<http://www.moh.gov.sg/cpg>.
31. The Taiwanese Osteoporosis Association. Taiwanese Guidelines for the Prevention and Treatment of Osteoporosis. Sept. 3, 2013.
32. College of Orthopaedic Surgeons of Thailand and Thai Osteoporosis Foundation. Clinical practice guideline for osteoporosis. 2010.
33. Hanoi, Viet Nam Education House. Diagnosis and Treatment of Osteoporosis. Guidelines for Diagnosis and Treatments of Common Rheumatic Diseases. 2012: 247-258.
34. Lau MC, Sambrook P, Seeman E, Leong KH, Leung PC and Delmas P. Guidelines for diagnosing, prevention and treatment of osteoporosis in Asia.
    Published in APLAR Journal of Rheumatology, Vol. 9, Issue 1, p. 24-26, May 2006:available at <http://www3.interscience.wiley.com/journal/120092309/issue>

**NORTH AMERICA**

**Canada**.

1. Papaioannou A, Morin S, Cheung AM, Atkinson S, Brown JP, Feldman S, et al. Scientific Advisory Council of Osteoporosis Canada.2010 clinical practice guidelines for the diagnosis and management of osteoporosis in Canada: summary. CMAJ. 2010 Nov 23;182(17):1864-1873.
2. Jaglal SB, Hawker G, Cameron C, Canavan J, Beaton D, Bogoch E, et al. Osteoporosis Research, Monitoring and Evaluation Working Group.[The Ontario Osteoporosis Strategy: implementation of a population-based osteoporosis action plan in Canada](http://www.ncbi.nlm.nih.gov/pubmed/20309525). Osteoporos Int. 2010 Jun;21(6):903-908.
3. Scott V, Duncan B. Clinical guidelines for seniors falls prevention. Vancouver: British Columbia Injury Research and Prevention Unit; 2006. Available from: <http://www.injuryresearch.bc.ca>.
4. Scott V. World Heath Organization Report: prevention of falls in older age. Falls Prevention: Policy, Research and Practice. 2007.
5. Lyons SS. Fall prevention for older adults: Evidence-based protocol. Research Translation and Dissemination Core; 2004.
6. Moreland J, Richardson J, Chan DH, O'Neill J, Bellissimo A, Grum RM, et al. Evidence based guidelines for the secondary prevention of falls in older adults. Gerontology. 2003; 49(2):93-116.
7. The Registered Nurses’ Association of Ontario. Preventing Falls and Reducing Injury from Falls (4th ed.). Toronto, 2017.
8. The Registered Nurses’ Association of Ontario. Preventing Falls and Reducing Injury from Falls (4th ed.). Toronto, 2005. [www.RNAO.ca/bpg](http://www.RNAO.ca/bpg).

**USA**

1. The National Osteoporosis Foundation (NOF) .Clinician's Guide to prevention and treatment of osteoporosis. 2014; 25(10): 2359–2381. doi:  10.1007/s00198-014-27942 <http://link.springer.com/article/10.1007%2Fs00198-014-2794-2>.
2. American Association of Clinical Endocrinologists and American College of Endocrinology Clinical Practice. Guidelines for the Diagnosis and Treatment of Postmenopausal Osteoporosis -2016
   <https://www.aace.com/files/final-appendix-sept-7.pdf>.
3. Preventive Services Task Force. Screening for osteoporosis: recommendation statement of the U.S. Annals of Internal Medicine 2011 Jan 18. Website:  <http://www.uspreventiveservicestaskforce.org/uspstf10/osteoporosis/osteors.htm>.
4. Management of osteoporosis in postmenopausal women: 2010 position statement of The North American Menopause Society
   <https://www.menopause.org/docs/default-document-library/psosteo10.pdf?sfvrsn=2>.
5. Grossman J M, Gordon R, Ranganath VK, Deal C, Caplan L, Chen W, et al. Recommendations for the prevention and treatment of glucocorticoid-induced osteoporosis. Arthritis Care Res, 62: 1515–1526. doi: 0.1002/acr.20295.
6. American Medical Directors Association. Falls and falls risk: clinical practice guideline. American Directors Association; 2003.
7. American Geriatrics Society, British Geriatrics Society, & American Academy of Orthopedic Surgeons Panel on Falls Prevention. Guidelines for the prevention of falls in older persons. J Am Geriatr Soc. 2001:49(5):664-72. [cited 2006 Oct 26] Available from:www.americangeriatrics.org/products/positionpapers/Falls.pdf

**MIDDLE EAST & NORTH AFRICA**

1. Pan Arab Osteoporosis Society. Guidelines for Osteoporosis Management . Mediterr J Rheumatol 2017; 28(1): 37-42 <http://www.mjrheum.org/march-2017/newsid792/71>.
2. Maalouf G, Gannage-yared MH, Ezzedine J, Larijani B, Badawi S, Rached S, et al. Middle East and North Africa Consensus on Osteoporosis.2008.
3. Meybodi HA, Heshmat R, Maasoumi Z, Soltani A, Hossein-Nezhad A, Keshtkar AA, Bahrami A, Rajabian R, Nabipour I, Omrani GH, Pajouhi M. Iranian osteoporosis research network: background, mission and its role in osteoporosis management. Iranian Journal of Public Health. 2008 Jan 1;37(supp):1-6.
4. Jordanian Osteoporosis Prevention Society. Osteoporosis Guideline,2017.p
5. El-Hajj Fuleihan G, Baddoura R, Awada H, Arabi A, and Okais J. First Update of the Lebanese Guidelines for Osteoporosis Assessment and Treatment (2007) J Med Liban 2007; 55(4):176-191.
6. El-Hajj Fuleihan G, Baddoura R, Awada H, Okais J, Risk P, McClung M. Lebanese guidelines on osteoporosis assessment and treatment. Journal of Clinical Densitometry (2004).
7. Chakhtoura M, Leslie WD, McClung M, Cheung AM, El-Hajj Fuleihan G. The FRAX-based Lebanese osteoporosis treatment guidelines: rationale for a hybrid model. Osteoporos Int (2017) 28:127–137 DOI 10.1007/s00198-016-3766-5.
8. Ezzahra Abourazzak F, Khazzani H, Mansouri S, Ali Ou Alla S, Allali F, El Maghraoui A, et al. Recommandations de la Société Marocaine de Rhumatologie sur la vitamine D chez l’Adulte. Rev Mar Rhum 2016; 35:3-15.
9. Al-Saleh Y, Sulimani R, Sabico S, Raef H, Fouda M, Alshahrani F, et al. Guidelines for osteoporosis in Saudi Arabia: recommendations from the Saudi Osteoporosis Society. Annals of Saudi medicine. 2015 Jan;35(1):1-2.
10. Hough S, Ascott-Evans BH, Brown SL, Cassim B, de Villiers TJ, Lipschitz S, et al. South African Clinical Guideline for the Diagnosis and Management of Osteoporosis. 2010 .

**LATIN AMERICA**

1. Schurman L, Bagur A, Hermberg HC, Messina OD, Negri AL, Sánchez A, et al. Guías 2012 para el diagnóstico, la prevención y el tratamiento de la osteoporosis. MEDICINA (Buenos Aires) 2013; 73: 55-74, ISSN 0025-7680.
2. Rodrigues Pereira RM, de Carvalho JF, Paula AP, Zerbini C et al. Guidelines for the prevention and treatment of glucorticoid-induced osteoporosis. Rev Braz Reumatol. 2012; 52(4):569-593.
3. The Brazilian Society of Endocrinology and Metabology (SBEM). Recommendations for the diagnosis and treatment of hypovitaminosis D .Arq Bras Endocrinol Metab. 2014;58/5.
4. Colombian Osteporosis Society. Management of Post-menopausal Osteoporosis. Revista Colombiana de Reumatología DOI: 10.1016/j.rcreu.2018.02.006.
